# Health Services Availability and Readiness for Management of Hypertension and Diabetes in Primary Care Health Facilities in Ghana: a Cardiovascular Risk Management project

**DOI:** 10.5334/gh.1375

**Published:** 2024-12-05

**Authors:** Thomas Hinneh, Bernard Mensah, Hosea Boakye, Oluwabunmi Ogungbe, Yvonne Commodore-Mensah

**Affiliations:** 1 Johns Hopkins School of Nursing, Baltimore, MD, USA; 2 Yale School of Nursing, New Haven, CT, USA; 3 Department of Physical Therapy, Boston University, Boston, MA, USA; 4 Johns Hopkins Bloomberg School of Public Health, Baltimore, MD, USA

**Keywords:** Cardiovascular diseases, hypertension, diabetes, Diabetes, Primary health care

## Abstract

**Introduction:**

Hypertension and diabetes are leading causes of adult hospital admissions and mortality across health facilities in Ghana. Timely screening and diagnosis at primary health facilities are crucial to initiate treatment and avert complications. This study explored service availability and readiness of health systems for managing hypertension and diabetes in selected district hospitals in Ghana.

**Methods:**

We adapted the World Health Organization (WHO) Service Availability and Readiness Assessment (SARA) tool to assess hypertension and diabetes management practices between June and July 2022 in four district hospitals in Ghana. Domain scores of service readiness were calculated based on the mean score of tracer item availability, transformed into percentages, and stratified by facility ownership. The mean readiness index was based on basic clinical logistics and equipment, diagnostic capacity, and first-line medications. Service availability was based on the core health workforce and specific service arrangements for the management of hypertension and diabetes. Facilities were considered ‘ready’ for services at a cut-off readiness score of 70%.

**Results:**

All facilities (n = 4, 100%) provided hypertension and diabetes services, with a median of 118 nurses (IQR 103–140) and 5 physicians (IQR 2–8). Only one facility (n = 1, 25%) had conducted cardiovascular disease training in the past year. All basic equipment (weighing scales, stethoscopes, glucometers, and blood pressure monitors) were available in all 4 facilities. Antihypertensives, including ACE inhibitors (n = 3; 75%), calcium channel blockers (n = 4; 100%), centrally acting agents (n = 4; 100%), and thiazides (n = 4; 100%), were available, as were antidiabetic medications like metformin (n = 4; 100%) and insulin (n = 2; 50%). Only two facilities (n = 2; 50%) could perform the required test (Hemoglobin A1c, full blood count, renal function, serum creatinine, blood urea, electrolytes, and blood lipid tests). Overall readiness score was 75.5%, essential medications (83.5%), basic equipment (78%), clinical guidelines for the management of cardiovascular disease management (75%), and diagnostic capacity (65.5%). Mission facilities had a higher readiness score (96%) and government facilities (55%).

**Conclusion:**

Facilities demonstrated high readiness for basic hypertension and diabetes care, with higher availability of some essential medications and basic clinical logistics and equipment. Limited diagnostic capacity and cardiovascular disease training, highlight areas of improvement to strengthen hypertension and diabetes services in Ghana.

## Introduction

Noncommunicable diseases (NCDs) have surpassed infectious diseases as the leading cause of mortality and morbidity globally (1,2). The World Health Organization (WHO) global target of reducing NCDs by 25% by 2025 may be unachieved in many low and middle-income countries (LMICs) due to the increasing burden of hypertension and diabetes, which are the common drivers of the global NCD burden (1). Out of the 1.4 billion people estimated to be living with hypertension worldwide, about two-thirds live in LMICs (3). According to the International Diabetes Federation (IDF), of 537 million adults with diabetes globally, three-fourths of them live in LMICs (4). Besides, hypertension and diabetes are common comorbidities that exacerbate the risk for cardiovascular and associated complications (5). The burden of these chronic conditions is projected to increase, and health systems in LMICs need to be well-resourced to effectively respond to this epidemiological shift (2,6). Yet, the economic burden of these conditions is substantial, with direct healthcare costs and loss of productivity placing significant strain on the already limited resources in Ghana (7,8). This financial strain not only impacts healthcare systems but also affects families and communities, leading to cycles of poverty and reduced economic growth.

In Ghana, about 34% of the population has hypertension (9). Nationwide data from the Ghana Health Service suggests that the burden of hypertension increased from 172,796 in 2018 to 193,099 in 2022, representing an increase of 11.74% within the four years. Within the same period, the burden of diabetes also increased from 617,563 to 622,849 (10). For diabetes, the marginal increase is likely an underestimation of the true burden of diabetes, given the low diagnosis rate, which is primarily due to the unavailability of laboratory tests to diagnose diabetes in some health facilities in Ghana (11). Additionally, there is no routine screening for diabetes, particularly during pregnancy (11,12). To this point, some studies have projected that almost half of adults with diabetes are undiagnosed in Ghana, which would cause an underestimation of the reported national burden (13,14,15).

Ghana has a pluralistic health systems arrangement for service delivery which includes mission, private, and government-owned facilities (16,17). These facilities share some commonalities in terms of health financing and compliance with guidelines for disease management (17). However, resource allocation, staff recruitment, monitoring and evaluation practices, and policies may differ. The Ministry of Health facilities have initiated capacity-building efforts such as media campaigns and community outreaches to enhance early detection and timely treatment to combat the rising burden of chronic diseases (16). However, very little is known about how institutional efforts are directed to screen, diagnose, and effectively and sustainably treat hypertension and diabetes cases. An understanding of service availability and readiness at primary health care level for hypertension and diabetes is crucial to informing strategies for efficient management to avert more morbidity, and mortality, and reduce the economic burden associated with NCDs (18).

Most clinical studies in Ghana have focused on quantifying the burden of hypertension and diabetes and related determinants for treatment outcomes which hinders the ability of policymakers, donors, and key actors to effectively target and allocate resources to support hypertension and diabetes care (18,19,20). This study aimed to assess the availability of services and readiness of district hospitals to manage hypertension and diabetes in Ghana.

## Methods

### Study design

This multicenter cross-sectional study was conducted between June 2023 and July 2023 in four districts in Ghana. The WHO-SARA questionnaire was modified to assess hypertension and diabetes services provision and was administered to key selected informants in the respective health facilities **(Supplementary file 1)**. The health facility SARA tool is designed to evaluate and monitor the availability and readiness of health services within a health system and uses a systematic survey approach to generate a set of tracer indicators for this purpose (21).

#### Study sites and population

**Figure 1:**
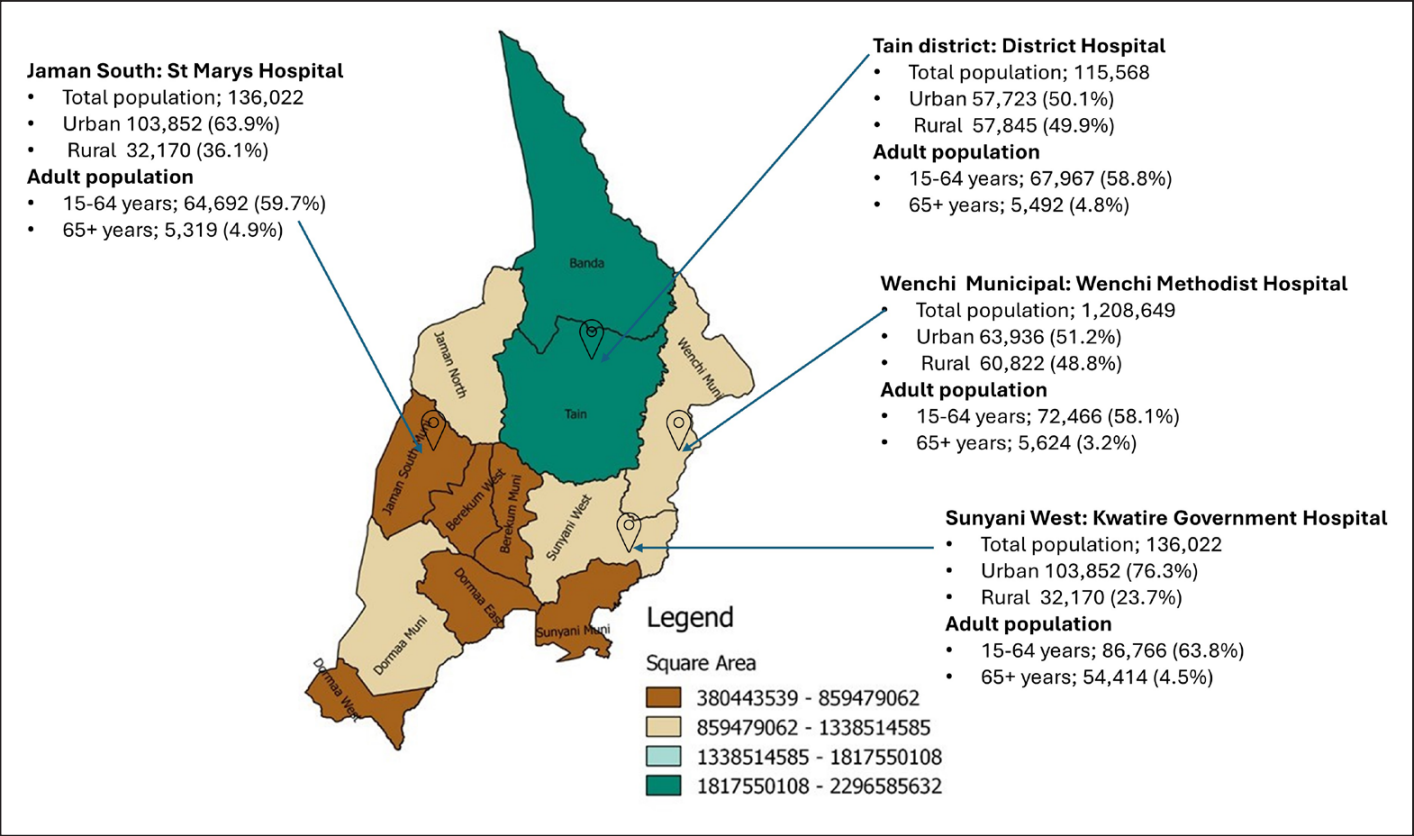
Map of the Bono Region showing the districts where the project was conducted. The percentage of the urban population in Bono Region was 58.6%. The four facilities are district hospital status selected across four district capitals: Source GSS 2021.

Two mission and two government facilities in the Bono of Ghana were selected as study sites with varying district demographic distribution as part of a pilot cardiovascular disease risk management project funded by a non-government organization. This study served as a preliminary assessment of the needs of the selected facilities. The secondary goal of this evaluation was to generate data to support health facilities and program funders in identifying gaps in hypertension and diabetes management in health facilities in Ghana. The Bono Region is one of the southern regions of Ghana, with an estimated population of 1,082,520, and was formerly part of the Brong Ahafo Region (22). The region has one regional hospital with 13 government district hospitals and five mission-based hospitals. Across health facilities in Ghana, hypertension and diabetes care are provided at designated district hospitals and secondary and tertiary level healthcare facilities. The district hospitals serve as the highest referral points for all primary health facilities in every district. The population demographics of the districts selected are presented in Figure 1.

#### Instrument adaptation

NCD domains of the SARA tool were adopted to assess health facilities’ availability and readiness to diagnose and treat hypertension and diabetes (21). Key terminologies are defined in Table 1. We adapted the components of the instrument that directly apply to NCD diagnosis, treatment, and management, specifically hypertension and diabetes service delivery. Items and modules that are not related to NCD management were removed from the survey. For instance, service availability questions on reproductive, maternal and newborn, child and adolescent health, and surgical services were not included. On service readiness, questions related to communications, ambulance/transportation for emergencies, power supply, infectious control precautions, and health care waste management were not included.

**Table 1:** Key definitions from the WHO SARA instrument.


**Service readiness:** Availability of the essential indicators for hypertension and diabetes care at the PHCs, including functional equipment, diagnostic capacity, national guidelines for hypertension and diabetes management, and first-line essential medications **First-line medications:** Listed essential hypertension and diabetes medications prescribed by the standard treatment guideline available with at least a 30-day stock **Supervision and monitoring:** Records of last facility monitoring, and supervisory visits from the higher level (pharmacy stocks, data completeness, and staffing) **Training/capacity building for hypertension/diabetes:** Records of training of healthcare workers on diagnosis and treatment of hypertension and diabetes in the last 12 months **Clinical guidelines for hypertension/diabetes management:** Available national guidelines for the diagnosis and management of hypertension/cardiovascular disease and diabetes and the standard treatment guideline at the time of assessment. **Service availability:** Hypertension/diabetes service availability was defined as the availability of diabetes and hypertension treatment services, including specialized service arrangements and the required staff to diagnose and manage hypertension/diabetes at district hospitals


Note: All definitions adopted from the WHO Service Readiness and Availability Analysis Guide.

#### Definition of key indicators

#### Data collection and management

Health information officers from respective health facilities underwent training on administering specific domains of the SARA tool. Each component of the instrument was administered to the designated healthcare worker cadre responsible for those functions. For example, the component on human resource amenities, training, and supervision was completed by health services administrators in consultation with medical directors and nurse administrators. Health information officers were required to have at least a BSc qualification and conducted weekly data reconciliation with the study team to ensure data completeness. Medication tally sheets and human resource files were inspected to ensure data accuracy and completeness.

#### Statistical analyses

Descriptive statistics were utilized to summarize domains using mean and standard deviation (SD) or median and interquartile range where applicable.

#### Service readiness

Service readiness was based on five domains: diagnostics capacity, first-line antihypertensives and diabetic medications, clinical hypertension/diabetes and guidelines, and basic functional clinical equipment, with over 13 hypertension and diabetes-specific tracer indicators (21). The domain score was estimated as a mean score based on the availability (available = 1, not available = 0) of each tracer item in each domain following the SARA analysis plan. For example, the domain score for basic equipment was estimated using the formulae:


\[
\text{Basic}~\text{equipment}~\text{score}~\left(\% \right)=\frac{\text{Number} ~\text{of} ~\text{avalbale} ~\text{functional} ~\text{equipment} ~ }{\text{Total} ~\text{number} ~\text{of} ~\text{tracer} ~\text{items} ~\text{in} ~\text{the} ~\text{domain}}~ \times \,\ 100\%
\]

\[
\text{Diagnostic}~\text{capacity}~\text{score}~\left(\% \right)=\frac{\text{Number} ~\text{of} ~\text{diagnostic} ~\text{test} ~\text{available} ~ }{\text{Total} ~\text{number} ~\text{of} ~\text{tracer} ~\text{items} ~\text{in} ~\text{the} ~\text{domain}}~ \times \,\ 100\%
\]

The overall service readiness score was estimated as the mean score of all domains expressed in percentage and presented using a bar chart. Domains for diagnostic services availability were presented using radar plots stratified by facility ownership to highlight the routine and baseline laboratory investigations prescribed for hypertension and diabetes management in line with standard treatment guidelines of the Ministry of Health, Ghana.

#### Service availability

Service availability was calculated based on the proportion of health facilities with available hypertension and diabetes care services including screening, diagnosis, treatment, and health workforce required based on the human resource norm of the Ghana Health Service. Core health workers considered in this analysis were pharmacists, physician assistants, nursing professionals, laboratory technicians, physicians, and community health nurses who were working full or part-time. Other service-specific arrangements such as hypertension clinics, and wellness clinics which were set up in compliance with the national NCD policy to augment NCD screening efforts, were assessed (23). A detailed formula and analysis plan is provided in Supplementary File 2. At a cut-off value of 70%, facilities were considered “ready” to manage hypertension and diabetes based on previous studies conducted in other LMICs (24,25,26). All analysis was conducted using STATA software version 18 and Microsoft Excel (27).

## Results

### Characteristics of the health facilities service availability

All health facilities were situated at the respective capitals of the districts, which were all urban settings (n = 4, 100%). All the facilities in this study provided the required hypertension and diabetes services, including screening, diagnosis, and treatment. Only one (n = 1, 25%) out of the four facilities had a designated space/clinic for hypertension and diabetes clinics, while the remaining facilities treated patients at general outpatient units. All the facilities (n = 4, 100%), had designated clinic days for hypertension and diabetes services. Additionally, two of the facilities (n = 2; 100%) had wellness clinics offering free screening for hypertension and diabetes, positioned at the entrance of health facilities and managed by non-physician health workers (nurses, nutrition officers, and public health nurses). Only one facility (n = 1, 25%) utilized electronic patient files; others relied on a manual paper records system. Cases managed at the facilities varied with higher burden in mission district hospitals over the 12 months (Table 2).

**Table 2:** Hypertension and diabetes services availability of the selected facilities.


CHARACTERISTICS	FREQUENCY N (%)

**Facility type**	

**Mission Hospitals**	2 (50%)

**Government facilities**	2 (50%)

**Location of Health facility** ^a^	

**Urban**	4 (100%)

**Designated space for hypertension clinic**	

**Yes**	1 (25%)

**Clinic day for hypertension services**	

**Yes**	4 (100%)

**Has wellness clinics for screening hypertension and diabetes** ^c^	

**Yes**	2 (50%)

**Hypertension and diabetes Services**	

**Diagnosis (On-site), n (%)**	4 (100%)

**Treatment (On-site), n (%)**	4 (100%)

**Health Information management systems**	

**Facility keeps patients records of patients’ visits, n (%)**	3 (75%)

**Facility keeps electronic patient files, n (%)**	1 (25%)

**Number of outpatient visits related to hypertension and diabetes over 12 months** ^b^	

**Hypertension cases reported**	866 (58%)

**Diabetes**	131 (9%)

**Both**	506 (34%)


^a^ All the facilities included in this study were district hospitals (highest level of primary health care), which served as referral points for health facilities in the respective districts. ^b^ Cases burden based on data extracted from patient records for the period January 2022 and December 2022. ^c^ Wellness clinics are established across health facilities in Ghana to provide free screening for non-communicable diseases.

### Human resources for the management of hypertension and diabetes

The staffing levels in the district hospitals included general physicians (median = 14; interquartile range [IQR] 3–8), specialist physicians (7, [IQR] 1–3), nursing professionals (118, [IQR] 103–140), and physician assistants (4, [IQR] 2–7). Additionally, there were midwifery professionals (40, [IQR] 40–45), pharmacists (2, [IQR] 2–2), community health nurses (13, [IQR] 11–17), and laboratory staff (12, [IQR] 9–15) (Table 3). While the number of nurses exceeded the Ghana health service requirement for district hospitals based on the adjusted workload category A, physicians were below the required number across all facilities. There was an adequate number of physician assistants, laboratory staff, and community health nurses (28). Only one (n = 1, 25%) facility had conducted training and capacity building for hypertension and diabetes, in the previous year. Half of the facilities (n = 2, 50%) had received supervision from the district health management team (DHMT) regarding cardiovascular disease management within the past three months. (Table 2). The monitoring and supervisory visit focused on staffing, pharmacy (stock level), and data (completeness, timeliness, and quality).

**Table 3:** Healthcare workers employed at selected district hospitals in Ghana, 2022.


STAFF CATEGORY	TOTAL	MEDIAN	IQR

**General Physicians**	14	5	(3–8)

**Specialist Physicians**	7	2	(1–3)

**Nursing professionals**	432	118	(103–140)

**Physician Assistants**	18	4	(2–7)

**Midwifery professionals**	178	40	(40–45)

**Pharmacists**	8	2	(2–2)

**Community health nurses**	62	13	(11–17)

**Laboratory staff**	48	12	(9–15)


IQR-interquartile range. Note: The required number of workers was deduced from the Ghana Health Service human resource norm based on the minimum requirement for facility classification with workload Category A.

#### Service readiness indices for hypertension and diabetes care, cardiovascular disease clinical guidelines, and basic clinical logistics and equipment

**Figure 2:**
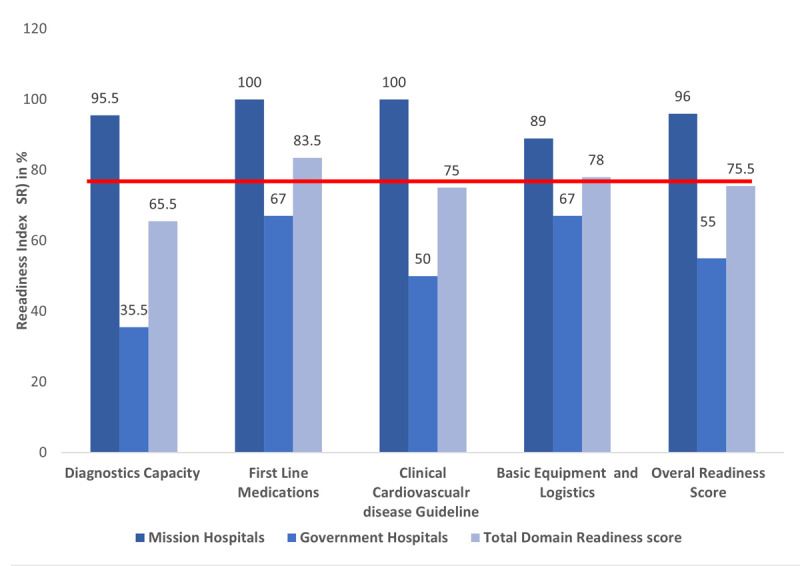
Readiness indices of hypertension and diabetes management at selected primary health facilities in Ghana, 2022.

Two of the mission locations (n = 2, 100%) and one of the government (n = 1, 50%) facilities had standard treatment guidelines and soft copies of clinical cardiovascular disease management. All facilities had adult weighing scales (n = 4, 100%), thermometers (n = 4, 100%), stethoscopes (n = 4, 100%), glucometers (n = 4, 100%), and at least one functional blood pressure apparatus (n = 4, 100%) in all clinical areas. Only two facilities had different cuff sizes for blood pressure devices ranging from pediatric to large size. The overall availability for clinical guidelines and basic clinical logistics and equipment were 75% and 78%, respectively. When contrasted by facility ownership, the readiness score of mission vs government facility for clinical guidelines (100% vs 50%), and basic clinical logistics and equipment was (89% vs 67%) (Figure 2).

#### First-line essential hypertension and diabetes medications

The availability of oral hypoglycemics and antihypertensive medications varied across health facilities and was relatively higher in mission than in government facilities: angiotensin-converting enzyme inhibitors (ACE-I) (n = 3; 75%), calcium channel blockers (n = 4; 100%), centrally acting agents (n = 4; 100%), and thiazides (n = 4; 100%). Regarding antidiabetic medications, all facilities had metformin (n = 4; 100%), insulin (n = 2, 50%); mission (n = 2, 50%), and government (0). Three facilities had aspirin (n = 3, 75%); of these, two were mission (n = 2, 100%), and one was government (n = 1, 50%). The overall medication domain score was higher in mission hospitals (100%) and government facilities (50%) (Figure 2).

#### Diagnostic capacity

**Figure 3:**
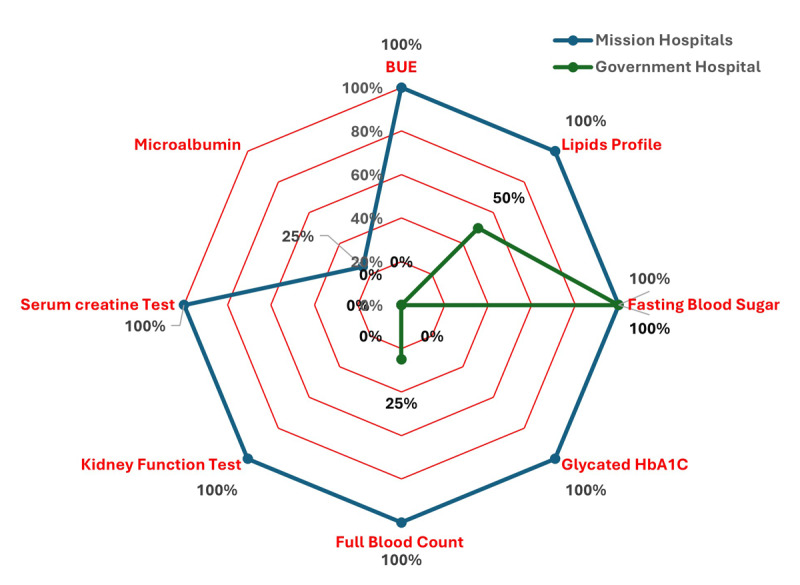
Radar plot of the diagnostic services availability in health facilities contrasted by facility ownership.

Only one facility (n = 1, 25%) had urine ketones and protein strips **(Supplementary File 3)**. All facilities could perform blood glucose tests (n = 4, 100%), although there were intermittent glucose strip shortages. Only two facilities were able to perform Hemoglobin A1c tests (n = 2, 50%), full blood count (n = 2, 50%), renal function (n = 2, 50%), serum creatinine (n = 2, 50%), blood urea, and electrolytes tests (BUE) (n = 2, 50%). Only two facilities (n = 2, 50%) were equipped to consistently conduct blood lipid tests (n = 2, 50%), a basic required test for diabetes management. The microalbumin test was available in only one facility (n = 1, 25%) (Figure 3). Both government facilities could not run liver function tests, serum creatine tests, renal function, blood lipid tests, microalbumin tests, and glycated hemoglobin (HbA1c), and often referred patients to external/private laboratory services. The overall mean readiness score for diagnostic capacity was lower in government hospitals (35.5%) than in mission hospitals (95.5%).

### Overall hypertension service availability and readiness score

Overall, hypertension and diabetes services were available in all facilities with wellness and nurse-led clinics set up to improve screening and linkage to care. Beside the limited number of physicians, all facilities had the required non-physician healthcare workers to support management of hypertension and diabetes. *Key observations made by data officers were the shortage of glucose stripes for a free screening of diabetes for patients at both hypertension clinics and diabetes clinics*. The average mean service readiness score was 75.5%; first-line essential antihypertensive and oral hypoglycemics 83.5%, basic equipment was 78%, clinical guidelines for the management of hypertension and diabetes was 75%, and diagnostic capacity 65.5%.

## Discussion

This study assesses the availability and readiness for hypertension and diabetes services in selected district hospitals to identify gaps in non-communicable disease control. Hypertension and diabetes services—which include screening, diagnosis, and treatment—were available across all health facilities. Gaps were identified in diagnostic services, the required number of physicians, essential medications, and opportunities for cardiovascular disease training for health workers. The overall mean readiness score was 75.5%, which is above the 70% threshold where facilities can be described as ‘ready’ to provide hypertension and diabetes services; gaps, however, differed by facility ownership. Comparably, the scores observed in this study are higher than earlier studies reported in LMICs (29,30).

Firstly, the observed number of physicians across the facilities was less than the required norm based on the workload category according to the Ghana Health Service Human Resource Norms for district hospitals (28). Perhaps, the impact of the limited number of physicians is mitigated by the higher number of nurses and other professionals who are delegated hypertension-specific tasks such as blood pressure measurement, patient follow-up, and healthy dietary and lifestyle counseling under the task-shifting policy in non-communicable disease response in Ghana (31,32). However, of concern is the limited frequency of hypertension and diabetes-specific training for other healthcare workers, which raises concern for hypertension and diabetes care. In this study, only one facility had physicians participate in any kind of training in the last year. While this study highlights the limited opportunities for capacity building among healthcare workers involved in hypertension and diabetes management, this shortfall may further hinder the effective adoption of task-shifting policies in non-communicable disease management and compromise the timely, high-quality delivery of hypertension and diabetes services in Ghana (33,34).

Further, the limited capacity for diagnostic services and lower availability of some diagnostics commodities observed in government facilities impacted the readiness score reported in this study. For instance, all the mission hospitals surveyed could perform all the mandatory tests for diabetes management, including kidney function tests, while these tests were almost unavailable in the government facilities. A key concern was the frequent shortage of reagents required to conduct these tests, despite the facilities having the necessary staff and infrastructure to perform them. Yet, these tests are the recommended tests for establishing diagnosis and routine management practice (35). This scenario may perhaps explain why case volume observed at mission hospitals over the period was higher than in government facilities. Moreover, patients preference and choice of care, may be influenced by the availability of stable diagnostic capacity and other service specific availability metrics (36). However, the higher diagnostic capacity of the mission facilities in this study needs to be interrogated further given that mission health facilities traditionally may have the leverage to charge extra costs to cover other clinical care activities (37).

Additionally, first-line antihypertensive and antidiabetic medications were mostly available in mission health facilities. For instance, aspirin and soluble glucose, which are not necessarily first-line antihypertensive and antidiabetic medications, were available in all mission hospitals, which was not the case in some of the government health facilities. While this study was not set to examine the number of medications vis a vis the patient volume across health facilities, the persistent availability and re-stocking of medications and reagents across mission health facilities requires further assessment to understand best practices of procurement and medication management. While resources are generally limited given the higher burden of hypertension and diabetes cases reported across health facilities in Ghana, weak supply chain systems, which may be attributed to late reimbursement of national health insurance scheme to health facilities, hinder the ability of health facilities to diagnose and manage hypertension and diabetes cases even in the presence of well-motivated and trained personnel (38).

Most health facilities had at least two function blood pressure devices across facilities. The data reviewers’ officers noted faulty devices including weighing scales that are yet to be repaired. Notably, none of the facilities had a documented repair or maintenance strategy for clinical devices, which may result in the wastage of resources. Also, blood pressure cuffs for use across different age groups were hardly available. This is problematic given that the available standard adult cuffs may not fit well with every patient, which may result in inaccurate readings and either underdiagnosis or overdiagnosis of hypertension (39).

### Strengths and limitations

Our study has several strengths. It is one of the few to document service availability and readiness of primary care health facilities to provide hypertension and diabetes services in Ghana. The use of the WHO-validated SARA instrument strengthened the validity of the results. Nevertheless, our study had certain limitations. The lack of a sampling frame due to the limited number of health facilities included in this study may limit the generalization of the study findings to other health facilities in Ghana. There is also an absence of a qualitative component of this study to provide a contextual explanation of the availability and readiness indices assessed in this study, particularly from the perspectives of healthcare workers. Additionally, the WHO-SARA tool was limited in capturing crucial facets of healthcare access, including geographical, service utilization, and quality-related aspects. Also, relying on self-reported questionnaires to gauge availability could have introduced potential errors and bias. Nonetheless, the facilities included provide services for people who live in both rural and urban contexts and across both mission and government-owned facilities, which are major healthcare blocs in Ghana. This enabled us to provide a snapshot scenario of service availability and readiness for hypertension and diabetes in Ghana.

## Conclusion

The findings of this study highlight the need to prioritize resource allocation to improve the delivery of hypertension and diabetes care in Ghana. Although efforts towards bridging the physician-population ratio over the last decade have been progressive, most facilities still have inadequate physicians. There is an opportunity to adopt and strengthen a team-based approach where other healthcare workers can perform advanced hypertension and diabetes tasks to mitigate the impact of physician shortage (40). Additionally, equipping health facilities with improved diagnostic services is critical for early case detection and timely management of hypertension and diabetes across health facilities in Ghana.

## Data Accessibility Statement

The datasets used and/or analyzed during the current study are available from the corresponding author upon reasonable request.

## Additional Files

The additional file for this article can be found as follows:

10.5334/gh.1375.s1 Supplementary File 1 Table. Modified WHO service availability and Readiness instrument.

10.5334/gh.1375.s2 Supplementary File 2 Table. Formula and analysis plan for service availability and readiness.

10.5334/gh.1375.s3 Supplementary File 3 Table. SARA Health facility results from four health facilities.

## Abbreviations

**NCD:** Noncommunicable Diseases

**SARA:** Service Availability and Readiness

**CVD:** Cardiovascular Diseases

**WHO:** World Health Organizations

**HCW(s):** Healthcare worker(s)

**DM:** Diabetes

**HTN:** Hypertension
