# Supplementary material for: Health Services Availability and Readiness for Management of Hypertension and Diabetes in Primary Care Health Facilities in Ghana: a Cardiovascular Risk Management project
Source: Glob Heart. 2024 Dec 5;19(1):92. doi: 10.5334/gh.1375 (PMC11623084; doi:10.5334/gh.1375)
Supplement: Supplementary File 1 Table. — Modified WHO service availability and Readiness instrument. [file gh-19-1-1375-s1.pdf]

Supplementary file 1: Modified WHO service availability and Readiness instrument

Number	Question	Result	
**SECTION 1: COVER PAGE**			
**INTERVIEWER VISITS**			
	Facility number		
	Is this a supervisor validation check of a facility?	DATA COLLECTION FOR FACILITY ASSESSMENT…………… SUPERVISOR VALIDATION……………………………………………	1 2

	1	2	3	FINAL VISIT
**Date** **Interviewer Name**				DAY MONTH YEAR INT. NUMBER

**FACILITY IDENTIFICATION**			
	Name of facility	______________________	
	Location of facility	______________________	
	Region/Province		
	District		
	Type of facility* * These should be adapted at country level prior to implementation*	NATIONAL REFERRAL HOSPITAL ……………………………… DISTRICT/PROVINCIAL HOSPITAL ……………………………… HEALTH CENTRE/CLINIC………………………………………… HEALTH POST………………………………………………… MATERNAL/CHILD HEALTH CLINIC …………………………… OTHER (SPECIFY) ______________________	1 2 3 4 5 96
	Managing Authority	GOVERNMENT/PUBLIC ………………………………………… NGO/NOT-FOR-PROFIT ……………………………………… PRIVATE-FOR-PROFIT………………………………………… MISSION/FAITH-	1 2 3 4 96

		BASED.. OTHER (SPECIFY) ________________________________	
	Urban/Rural	URBAN<br>..<br>RURAL<br>...	1<br>2
	Outpatient only	YES<br>...<br>NO...	1<br>2

Number	Question	Result
**<u>GEOGRAPHIC COORDINATES</u>**		
**COLLECT GEOGRAPHIC COORDINATES INFORMATION FOLLOWING THE INSTRUCTIONS*. SET DEFAULT SETTINGS FOR GPS:**<br>1. **SET COORDINATE FORMAT TO DECIMAL DEGREES (HDDD.DDDDD)**<br>2. **SET “DATUM” TO WGS84**<br>3. **SET “UNITS” TO METRIC, “NORTH REF” TO MAGNETIC AND “ANGLE” TO DEGREE**<br><br>**MOVE TO MAIN ENTRANCE OF THE BUILDING. STAND WITHIN 30 METERS OF DOOR WHERE ENTRANCE IS IN PLAIN VIEW TO THE SKY.**<br><br>1. **TURN GPS RECEIVER ON AND WAIT UNTIL SATELLITE PAGE INDICATES "READY TO NAVIGATE" AND ACCURACY IS AT A RECOMMANDED LEVEL**<br>2. **GO TO THE “MENU” PAGE AND SELECT "MARK"**<br>3. **HIGHLIGHT THE WAYPOINT NUMBER AND PRESS "ENTER"**<br>4. **ENTER FACILITY CODE AND PRESS “ENTER” TO GO BACK TO THE “MARK” PAGE**<br>5. **HIGHLIGHT "OK" AND PRESS "ENTER" TO REGISTER THE WAYPOINT**<br>6. **GO TO THE MENU PAGE, HIGHLIGHT "WAYPOINT" AND PRESS "ENTER"**<br>7. **HIGHLIGHT THE WAYPOINT AND PRESS “ENTER” TO OPEN ITS DETAILED INFORMATION**<br>8. **COPY INFORMATION FROM WAYPOINT LIST PAGE IN THE FORM BELOW**<br><br>**BE SURE TO COPY THE WAYPOINT NAME (FACILITY NUMBER) FROM THE WAYPOINT LIST PAGE TO VERIFY THAT YOU ARE ENTERING THE CORRECT WAYPOINT INFORMATION ON THE DATA FORM**		
	Waypoint name (Facility number)	[ ][ ][ ][ ][ ][ ]
	Altitude	[ ][ ][ ][ ] Meters
	Latitude	N/S………… a ……<br><br>DEGREES/ DEC b [ ][ ] . c [ ][ ][ ][ ][ ]
	Longitude	E/W .... a<br><br>DEGREES/DE [ ][ ][ ] . c [ ][ ][ ][ ][ ]

		C b		

Num	Number	Question	Result					
	SUPERVISION							
	01	When was the last time this facility received a supervision visit from the higher level CONCERNING NCDs (DHMT or other)?	THIS MONTH ....1<br>IN THE LAST 3 MONTHS ....2<br>MORE THAN 3 MONTHS AGO...........3<br>DON'T KNOW..................................98					
	02	During the supervision visit, did the supervisor assess the following?	**YES**<br>**1**			**NO**<br>**2**		
	03	Pharmacy (e.g. drug stock out, expiry, records, etc.)						
	04	Staffing (e.g. staff availability and training)						
	05	Data (e.g. completeness, quality, and timely reporting)						
	GENERAL OUTPATIENT SECTION							
	BASIC EQUIPMENT							
	500	Please tell me if the following basic equipment and supplies used in the provision of client services are available and functional in this facility today.<br>**ASK TO SEE THE ITEM**	**A) AVAILABLE**			**B) FUNCTIONING**		
			OBSERVED<br>1	REPORTED NOT SEEN<br>2	NOT AVAILABLE<br>3	YES<br>1	NO<br>2	DON'T KNOW<br>8
	01	Adult weighing scale						
	02	Glucometer test strips (with valid expiration date)						
	03	Glucometer						
	04	Measuring tape-height board/stadiometer						
	05	Thermometer						
	06	Urine protein strip						
	07	Stethoscope						

	08	Blood pressure apparatus (may be digital or manual sphygmomanometer with a stethoscope)						
	09	Urine ketones strip						

Indicator Number code		Question	Result		Skip
	MODULE 1: SERVICE AVAILABILITY				
	SECTION 2: STAFFING				
		I have a few questions on staffing for this facility. Please tell me how many staff with each of the following qualifications are currently assigned to, employed by, or seconded to this facility. Please count each staff member only once, based on the highest technical or professional qualification. For doctors, I would also like to know, of the total number, how many are part-time in this facility.	**A) ASSIGNED/ EMPLOYED/ SECONDED (INCLUDING PART TIME)**	**B) PART TIME**	
	01	Generalist (non-specialist) medical doctors			
	02	Specialist medical doctors			
	03	Medical Drs and PAs/			
	04	Nursing professionals			
	06	Pharmacists			
	07	Laboratory technicians (medical and pathology)			
	08	Community health nurses			

	D. NON-COMMUNICABLE DISEASES			
	**2000**	Does this facility offer diagnosis or management of non-communicable diseases, such as diabetes, cardiovascular disease,	YES 1<br>NO 2	
	**2001**	Do providers in this facility diagnose and/or manage diabetes in patients?	YES .... 1<br>NO 2	
	**2002**	Do you have the national guidelines for the diagnosis and management of diabetes available in this facility today?<br>**IF AVAILABLE, ASK TO SEE THE DOCUMENT**	YES, OBSERVED .... 1<br>YES, REPORTED NOT SEEN .... 2<br>NO 3	
	**2003**	Have you or any provider(s) of diabetes services received any training in the diagnosis and management of diabetes in the last two years?	YES .... 1<br>NO 2	
	**2004**	Do providers in this facility diagnose and/or manage cardiovascular diseases such as hypertension in patients?	YES .... 1<br>NO 2	S23
	**2005**	Do you have the national guidelines for the diagnosis and management of cardiovascular diseases available in this facility today?<br>**IF AVAILABLE, ASK TO SEE THE DOCUMENT**	YES, OBSERVED .... 1<br>YES, REPORTED NOT SEEN .... 2<br>NO 3	
	**2006**	Have you or any provider(s) of services for cardiovascular diseases received any training in the diagnosis and management of cardiovascular diseases such as hypertension in the last two years?	YES .... 1<br>NO 2	

Indicator code	Number	Question	Result		Skip
	SECTION 6: DIAGNOSTICS				
	ASK TO BE SHOWN THE MAIN LABORATORY OR LOCATION IN THE FACILITY WHERE MOST TESTING IS DONE TO START DATA COLLECTION. INTRODUCE YOURSELF AND EXPLAIN THE PURPOSE OF THE SURVEY, THEN ASK THE FOLLOWING QUESTIONS.				
	I would like to know if the following diagnostic tests and associated equipment are available today in this facility.				
	3001	Does this facility offer any of the following tests on-site?	**YES (ONSITE)**<br>**1**	**NO**<br>**2**	
	3002	Blood glucose tests using a glucometer			
	3003	Serum creatinine testing			
	3004	Other liver function testing (such as bilirubin)			
	3005	Other renal function testing (such as urea nitrogen)			
	3006	Does this facility do full blood count and differential testing onsite or offsite?	YES, ONSITE ....1<br>YES, OFFSITE ....2<br>NO 3		
	3007	Does this facility do full blood count and differential testing onsite or offsite?	YES, ONSITE ....1<br>YES, OFFSITE ....2<br>NO 3		
	3008	Blood urea, electrolytes and creatinine BUE			
	3009	Glycated haemoglobin (HbA1c)			
	3010	Fasting blood lipid profile (adults)<br>Urine microalbumin			

Indicator code	Number	Question	Result					Skip
	SECTION 7: MEDICINES AND COMMODITIES							
	**ASK TO BE SHOWN THE MAIN LOCATION IN THE FACILITY WHERE MEDICINES AND OTHER SUPPLIES ARE STORED. FIND THE PERSON MOST KNOWLEDGEABLE ABOUT STORAGE AND MANAGEMENT OF MEDICINES AND SUPPLIES IN THE FACILITY. INTRODUCE YOURSELF, EXPLAIN THE PURPOSE OF THE SURVEY AND ASK THE FOLLOWING QUESTIONS.**							
	**I would like to know if the following medicines are available today in this facility. I would also like to observe the medicines that are available. If any of the medicines I mention are stored in another location in the facility, please tell me where in the facility it is stored so I can go there to verify.**							
		Are any of the following medicines for the management of **non-communicable diseases** available in the facility today? **CHECK TO SEE IF AT LEAST ONE OF EACH MEDICINE IS VALID (NOT EXPIRED)**	**OBSERVED AVAILABLE**		**NOT OBSERVED**			
			AT LEAST ONE VALID 1	AVAILABLE NON VALID 2	REPORTED AVAILABLE BUT NOT SEEN 3	NOT AVAILABLE TODAY 4	NEVER AVAILABLE 5	
	01	Metformin cap/tab						
	02	Insulin regular injection						
	03	Glucose 50% injection						
	04	ACE inhibitor (e.g. enalapril, lisinopril, ramipril, perindopril)						
	05	Thiazide (e.g. hydrochlorothiazide)						
	06	Beta blocker (e.g. Bisoprolol, metoprolol, carvedilol, atenolol)						
	07	Calcium channel blocker (e.g. amlodipine)						
	08	Aspirin cap/tab						
	09	Lisinopril						

Number	Question	Result	Skip
**SECTION 8: INTERVIEWER'S OBSERVATIONS**			
	**INTERVIEW END TIME** (use the 24 hour-clock system)	:	
	**RESULT CODES (LAST VISIT):**	COMPLETED.................................... 1<br>RESPONDENT NOT AVAILABLE ...... 2<br>REFUSED.. 3<br>PARTIALLY COMPLETED .... 4<br>OTHER ______________________ 96<br>(SPECIFY)	

COMMENTS ABOUT THE RESPONDENT:

____
____
____
____

COMMENTS ON SPECIFIC QUESTIONS:

____
____
____
____
____

ANY OTHER COMMENTS:

____
____
____

SUPERVISOR'S OBSERVATIONS:

____

**NAME OF SUPERVISOR:** ______________________	DATE: ______________
