# Supplementary material for: Health Services Availability and Readiness for Management of Hypertension and Diabetes in Primary Care Health Facilities in Ghana: a Cardiovascular Risk Management project
Source: Glob Heart. 2024 Dec 5;19(1):92. doi: 10.5334/gh.1375 (PMC11623084; doi:10.5334/gh.1375)
Supplement: Supplementary File 2 Table. — Formula and analysis plan for service availability and readiness. [file gh-19-1-1375-s2.pdf]

Supplementary file Table 2: Formula and analysis plan for service availability and readiness

Index	Domain	Tracer items	Domain score (Mean availability of items)
Service-specific availability	Health workforce	Generalist (non-specialist)	Descriptive (Based on Ghana Health Service Human resource norm
		Specialist medical doctors	
		Medical Drs and PAs/	
		Nursing professionals	
		Pharmacists	
		Laboratory technicians	
	DM/HTN screening and management services	DM screening and management practices	Descriptive summary
		HTN screening and management services	
	HTN and DM-specific service provision	Clinic space and designated days	Descriptive summary
		Wellness clinics	
Service Readiness	Clinical guideline	Availability of national guidelines for the diagnosis and management of diabetes	n / 2× 100 where n is the total number of items available in the domain
		Availability of national guidelines for the diagnosis and management of CVD?	
	Basic equipment	Adult weighing scale	n / 9× 100 where n is the total number of items available in the domain
		Measuring tape-height board/stadiometer	
		Thermometer	
		Urine protein strip	
		Glucometer test strips (with valid expiration date)	
		Stethoscope	
		Glucometer	
		Blood pressure apparatus (may be digital or manual sphygmomanometer with a stethoscope)	
		Urine Ketones strip	
	Diagnostic capacity	Blood glucose tests using a glucometer	n / 8× 100 where n is the total number of items available in the domain
		Serum creatinine testing	
		Other liver function testing (such as bilirubin)	

		Other renal function testing (such as urea nitrogen)	
		Does this facility do full blood count and differential testing onsite or offsite?	
		Does this facility do full blood count and differential testing onsite or offsite?	
		Blood urea, electrolytes and creatinine BUE	
		Glycated haemoglobin (HbA1c)	
	Medicine and commodities	Metformin cap/tab	n / 9 × 100 where n is the total number of items available in the domain
		Insulin regular injection	
		Glucose 50% injection	
		ACE inhibitor (e.g. enalapril, lisinopril, ramipril, perindopril)	
		Thiazide (e.g. hydrochlorothiazide)	
		Beta blocker (e.g. Bisoprolol, metoprolol, carvedilol, atenolol)	
		Calcium channel blocker (e.g. amlodipine)	
		Aspirin cap/tab	
HTN-hypertension<br>DM -diabetes			
