# Supplementary material for: Health Services Availability and Readiness for Management of Hypertension and Diabetes in Primary Care Health Facilities in Ghana: a Cardiovascular Risk Management project
Source: Glob Heart. 2024 Dec 5;19(1):92. doi: 10.5334/gh.1375 (PMC11623084; doi:10.5334/gh.1375)
Supplement: Supplementary File 3 Table. — SARA Health facility results from four health facilities. [file gh-19-1-1375-s3.pdf]

Supplementary file 3 Table: SARA Health facility results from 4 health facilities in Bono Region Ghana

Tracer	Total number of facilities (n=4)	Mission hospitals (n=2)	Government hospitals (n=2)
**A. FACILITY SERVICES**			
**Diagnosis or management of**			
Hypertension	**4 (100%)**	2 (n=50%)	2 (n=50%)
Diabetes	**4 (100%)**	2 (n=50%)	2 (n=50%)
**B. MONITORING AND SUPERVISORY VISITS**			
Supervision visit from the higher level about NCDs (DHMT or other) in the last 3 months?	2 (50%)	1 (n=50)	1 (50%)
Pharmacy (e.g., drug stock out, expiry, records, etc.)	2 (n=50%)	1 (n=50%)	1 (50%)
Staffing (e.g., Staff availability and training)	2 (n=50%)	1 (n=50%)	1 (50%)
Data (e.g., completeness, quality, and timely	1 (n=25%)	1 (50%)	1 (n=50%)
**C. STAFFING**			
Generalist (non-specialist) medical doctors	14	3	11
Specialist medical doctors	7	6	1
Physician Assistants	18	15	3
Laboratory staff	48	24	24
Community health nurses	178	36	36
Nursing professionals	432	229	209
Midwifery professionals	178	100	78
Pharmacists	8	4	4
Staff training			
Trained on diagnosis and management of HTN/CVD	1 (n=25%)	0	1 (n=50%)
Trained on diagnosis and management of DM	0	0	0
**D. BASIC EQUIPMENT**			
Adult weighing scale	4 (100%)	2 (n=100%)	2 (n=100%)
Glucometer test strips (with valid expiration	3 (75%)	2 (n=100%)	1 (n=50%)
Glucometer	4 (100%)	2 (n=100%)	2 (n=100%)
Urine protein strip	1 (n=25%)	1 (n=50%)	0
Urine ketones strip	1 (n=25%)	1 (n=50%)	0
Measuring tape-height board/stadiometer	3 (75%)	2 (n=100%)	1 (n=50%)
Thermometer	4 (100%)	2 (n=100%)	2 (n=100%)

stethoscope	4 (100%)	2 (n=100%)	2 (n=100%)
BP Apparatus	4 (100%)	2 (100%)	2 (100%)
**E. CLINICAL GUIDELINES**			
National guidelines for HTN/DM management [38]	3 (75%)	2 (100%)	1 (50%)
**F. CVD AND DM 1ST LINE MEDICATIONS**			
Metformin cap/tab	4 (100%)	2 (100%)	2 (100%)
Insulin regular injection	2 (50%)	2 (100%)	0
Glucose 50% injection	2 (50%)	2 100%)	0
ACE inhibitor (e.g. enalapril, lisinopril, ramipril, perindopril)	3 (75%)	2 (100%)	1 (50%)
Thiazide (e.g. hydrochlorothiazide)	4 (100%)	2 (100%)	2 (100%)
Beta-blockers (e.g. Bisoprolol, metoprolol, carvedilol, atenolol)	4 (100%)	2 (100%)	2 (100%)
Calcium channel blocker (e.g. amlodipine)	4 (100%)	2 (100%)	2 (100%)
Aspirin cap/tab	3 (75%)	2 (100%)	1 (50%)
Lisinopril	4 (100%)	2 (100%)	2 (100%)
**G. DIAGNOSTIC CAPACITY**			
*Blood glucose tests using a glucometer	4 (100%)	2 (100%)	2 (100%)
*Serum creatinine testing	2 (50%)	2 100%)	0
Other liver function testing (such as bilirubin)	2 (50%)	2 100%)	0
*Other renal function testing	2 (50%)	2 100%)	0
*Full blood counts	4 (100%)	2 (100%)	2 (100%)
Does this facility do a full blood count onsite	3 (75%)	2 (100%)	1 (50%)
*Blood urea, electrolytes and creatinine BUE	2 (50%)	2 (100%)	0
*Glycated haemoglobin (HbA1c)	2 (50%)	2 (100%)	0
*Blood lipid profile (adults)	2 (50%)	2 (100%)	0
*microalbumin	1 (25%)	1 (50%)	0

HTN-Hypertension
DM- Diabetes
Cardiovascular diseases
*Routine investigation during diagnosis and follow-up for diabetes management -Ghana Health Service Standard Treatment Guideline[38]
